# Interleukin-1 Family Cytokines: Keystones in Liver Inflammatory Diseases

**DOI:** 10.3389/fimmu.2019.02014

**Published:** 2019-08-27

**Authors:** Louise Barbier, Maroua Ferhat, Ephrem Salamé, Aurélie Robin, André Herbelin, Jean-Marc Gombert, Christine Silvain, Alice Barbarin

**Affiliations:** ^1^INSERM U1082, Poitiers, France; ^2^Department of Digestive Surgery and Liver Transplantation, Trousseau University Hospital, Tours University, Tours, France; ^3^INSERM U1082, Poitiers University, Poitiers, France; ^4^INSERM U1082, Poitiers University Hospital, Poitiers, France; ^5^Department of Immunology and Inflammation, Poitiers University Hospital, University of Poitiers, Poitiers, France; ^6^Department of Hepatology and Gastroenterology, Poitiers University Hospital, University of Poitiers, Poitiers, France

**Keywords:** interleukin-1 family cytokines, liver diseases, inflammation, innate immunity, trained immunity, invariant natural killer T-cells, natural killer cells, innate lymphoid cells

## Abstract

The pyrogenic property being the first activity described, members of the interleukin-1 superfamily (IL-1α, IL-1β, IL-18, and the newest members: IL-33, IL-36, IL-37, and IL-38) are now known to be involved in several inflammatory diseases such as obesity, atherosclerosis, cancer, viral and parasite infections, and auto-inflammatory syndromes as well as liver diseases. Inflammation processes are keystones of chronic liver diseases, of which the etiology may be viral or toxic, as in alcoholic or non-alcoholic liver diseases. Inflammation is also at stake in acute liver failure involving massive necrosis, and in ischemia-reperfusion injury in the setting of liver transplantation. The role of the IL-1 superfamily of cytokines and receptors in liver diseases can be either protective or pro-inflammatory, depending on timing and the environment. Our review provides an overview of current understanding of the IL-1 family members in liver inflammation, highlighting recent key investigations, and therapeutic perspectives. We have tried to apply the concept of trained immunity to liver diseases, based on the role of the members of the IL-1 superfamily, first of all IL-1β but also IL-18 and IL-33, in modulating innate lymphoid immunity carried by natural killer cells, innate lymphoid cells or innate T-αβ lymphocytes.

## The Expanding Family of Interleukin (IL)-1 Cytokines and Their Receptors

### From “Endogenous Pyrogens” to the IL-1 Superfamily of Cytokines

The story of the IL-1 superfamily of cytokines began in the 1940s when Menkin (1), Beeson (2), and then Atkins (3) described a group of proteins, named “endogenous pyrogens,” responsible for fever, produced by leucocytes, and able to mediate multiple biological activities (4–6). By the late 1970s, the term “interleukin” was being used to describe the pleiotropic factors implicated in inflammatory immune response and the designation “IL-1” was then used to define the factors secreted by macrophages (4, 6). The discovery of IL-1, and its two forms IL-1α and IL-1β, is considered the birth of “cytokine biology,” and it led to the discovery of more members. This is how, over the years, IL-18, IL-33, IL-36, IL-37, and IL-38, which share several functional and structural properties, have been added to and included in the IL-1 superfamily of cytokines.

### IL-1 Sub-Families

IL-1 superfamily (7) is divided into three sub-families according to the length of the N-term pro-pieces: IL-1 sub-family (IL-1α, IL-1β and IL-33, IL-1Ra), IL-18 sub-family (IL-18 and IL-37), and IL-36 sub-family (IL-36 α, β, γ, and IL-38) (Table 1). Among these cytokines, in each sub-family one may distinguish “pro-inflammatory” members such as IL-1α, IL-1β, IL-33 or IL-18 from “anti-inflammatory” members such as IL-1Ra, IL-36Ra, IL-37, or IL-38 [Table 1, and for reviews: (7–9)].

**Table 1:** IL-1 cytokine superfamily characteristics.

**Cytokine name**	**Specific receptor chain name**	**Alternative receptor name**	**Co-receptor**	**Alternative co-receptor name**	**Function**	**Inhibitory ligand/Interference ligand**
IL-1α, IL-1β	IL-1R1	CD121a	IL-1R3	IL-1RAcP	Pro-inflammatory	IL-1Ra, sIL-1R1
IL-1β	IL-1R2	CD121b	IL-1R3	IL-1RAcP	Anti-inflammatory	sIL-1R2
IL-18	IL-1R5	IL-18Rα, IL-2Rrp, CD218a	IL-1R7, and/or IL-R8 (inhibitory)	IL-18Rβ, IL-18RAcP, CD218b	Pro-inflammatory	IL-18BP
IL-33	IL-1R4	T1/ST2, ST2L, IL-1RL1, IL-33R	IL-1R3 and/or IL-R8 (inhibitory)	IL-1RAcP	Pro-inflammatory	sST2 (sIL1R4)
IL-36α, IL-36β, IL-36γ	IL-1R6	IL-1Rrp2, IL-1RL2, IL-36R	IL-1R3	IL-1RAcP	Pro-inflammatory	IL-36Ra
IL-36Ra	IL-1R6	IL-1Rrp2, IL-1RL2, IL-36R	IL-1R3	IL-1RAcP	Anti-inflammatory	
IL-37	IL-1R5	IL-18Rα, IL-2Rrp, CD218a	IL-1R8	TIR8, SIGIRR	Anti-inflammatory	
IL-38	IL-1R6	IL-1Rrp2, IL-1RL2, IL-36R	IL-1R9	IL-1RAPL, TIGIRR-2	Anti-inflammatory	
Not known	NA		IL-1R8	TIR8, SIGIRR	Anti-inflammatory	
Not known	NA		IL-1R9 and IL-1R10	IL-1RAPL, TIGIRR-2 TIGIRR, TIGIRR-1	Not known	

*IL-1RAcP, Interleukin-1 Receptor Associated Protein; IL-1RAPL, Interleukin-1 Associated Protein Like 1/2; IL-1Ra, Interleukin-1 antagonist; IL-18BP, IL-18 Binding Protein; SIGIRR, Single Ig Interleukin-1 Related Receptor; TIGIRR, Three Ig Interleukin-1 Related Receptor; NA, not applicable*.

All of their receptors are heteropolymers of which at least one sub-unit is a member of the family of IL-1 receptors (IL-1R), which are characterized by extracellular immunoglobulin-like domains and an intracellular Toll/Interleukin-1R (TIR) domain within their cytoplasmic tail. Upon cell stimulation, IL-1R sub-units are oligomerized through the TIR domains. Secondly, MyD88 binds to the TIR domain, triggering nuclear factor-κB (NF-κB) translocation to the nucleus and activation of mitogen-activated protein kinases (MAPK) such as p38 and JNK, thereby leading to pro-inflammatory responses.

The combination of receptor sub-units conditions the type of signal transduced. For example, while association of IL-1RAcP (chain common to IL-1 and IL-1R3 receptors in the new nomenclature; Table 1) with the IL-1R1 sub-unit transduces an activation signal following binding of IL-1α or IL-1β, association of IL-1RAcP with IL-1R2 constitutes an IL-1β receptor that transduces an inhibitory signal.

IL-1RAcP is also a sub-unit of the IL-33 and IL-36 receptors, which associate IL-1RAcP with the IL-1RL1 chain (also called ST2: “suppression of tumorigenicity 2”, T1/ST2 or IL-1-R4) and with the IL-1Rrp chain (also called IL-36 R or IL-1R6), respectively.

IL-1R8 (SIGIRR for Single Ig IL-1 related receptor), another receptor of the IL-1 family, has no known ligands but transduces an inhibitory signal and has been described as a checkpoint for terminal maturation and acquisition of the effector functions of NK cells (10). Mantovani et al. have suggested that IL-1R8 interferes with the TIR-domain oligomerization of the IL-1 family receptors engaged by a ligand agonist, thereby leading to blocked recruitment of the MyD88 adapter (9, 11). Lastly, IL-1R8 is a sub-unit of the IL-37 receptor. IL-37 can reduce production of the pro-inflammatory cytokines IL-1α and IL-1β, IL-1Ra, IL-8, IL-17, IL-23, tumor necrosis factor (TNFα), and interferon γ (IFNγ) through IL-18Rα and IL-1R8 (SIGIRR) (12).

It is important to note that there exist soluble forms of the IL-1, sIL-1R1, and sIL-1R2 receptors that possess the inhibitory functions of IL-1. On the same token, IL-18BP (Binding Protein), which consists in a single IgG domain such as the transmembrane receptor IL-1R8, is a soluble factor interfering with the liaison of IL-18 and its receptor. IL-18BP possesses a particularly strong affinity with IL-18. Seric concentrations of IL-18BP in a healthy individual generally range from 2,000 to 4,000 pg/ml, which means that in steady state circulation, there is always a major excess of IL-18BP compared to IL-18. In a pathological situation, however, the anti-inflammatory effects of IL-18BP are liable to be attenuated due to the fact that IL-18BP likewise binds to IL-37. While blocking the anti-inflammatory functions of IL-37, this association is liable to render excessive the active free form of IL-18.

As regards the IL-33 receptor, there exists a soluble free form of the chain specific to the ST2 receptor, sST2. It is derived from alternative splicing of the ST2 mRNA (13). The biological relevance of the sST2 chain has remained elusive: Is it, as suggested in some studies, a decoy receptor enabling IL-33 neutralization, or is it simply the signature of the bringing into play of the IL-33/ST2 axis, with the seric sST2 concentrations being correlated with the severity of the pathology under consideration (13–16).

All in all, these different elements demonstrate the existence of cross-regulation between the different IL-1 family cytokines, of which each member possesses a counter-regulatory receptor or ligand.

### IL-1 Superfamily Members Differentially Depend on Processing Mechanisms to Function

IL-1 superfamily members (cytokines and receptors) contribute to a wide spectrum of immunological and inflammatory responses. Most of their members lack a signal peptide and are not immediately secreted. They are found diffusely in the cytoplasm as precursors containing cleavage sites and the difference that exists between members in terms of expression and proteolytic processing impact their respective roles *in vivo*. Indeed, to be biologically active, full-length precursors of IL-1β, IL-18, and IL-37 require intracellular processing, which depends on caspase-1, and the activation of the NOD-like receptor family, pyrin domain containing 3 (NLRP3)-inflammasome, required for conversion of the procaspase-1 into the active caspase-1. While pro-IL-36 and pro-IL-38 also need to be cleaved to generate a mature active form, the underlying mechanism does not depend on caspase-1 and remains unclear.

Remarkably, IL-1α and IL-33 are considered dual-function cytokines, meaning that in addition to their function as a classical cytokine, full-length IL-1α and full-length IL-33 (as part of damage-associated molecular pattern or DAMP) act as an “alarmin.” IL-1α and IL-33 precursors are constitutively expressed in the nuclei of epithelial and endothelial cells from different organs such as kidneys, liver, and lung. Moreover, the nuclear location of IL-33 seems to correspond to a storage function, a supposition confirmed by observation showing that mice expressing IL-33 from which the nuclear anchoring area has been eliminated have a fatal systemic inflammatory response syndrome, which is due to the absence of cellular retention of IL-33 (17). As regards IL-1α, a steady-state shuttle of cytokines between nucleus and cytoplasm has been described (18). In the event of a pro-apoptotic signal, IL-1α leaves the cytoplasm, is closely bound with the chromatin complex, and has no pro-inflammatory biological activity. By contrast, in the event of a necrotic signal, IL-1α leaves the nucleus and moves toward the cytoplasm, as the necrotic cell releases pro-inflammatory IL-1α (18). To this day no similar phenomenon has been documented regarding IL-33.

Consequent to tissue damage, biologically active full-length IL-1α and IL-33 are rapidly released by necrotic cells to alert the immune system to the danger, resulting in rapid production of pro-inflammatory mediators and infiltration of polymorphonuclear neutrophils (PMN) initially followed by monocytes/macrophages to the insult site.

**Figure 1:**
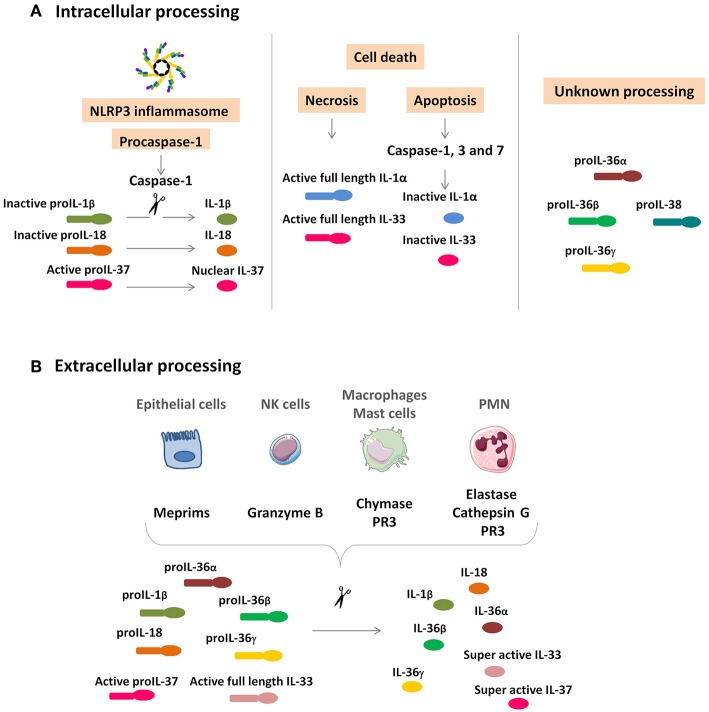
IL-1 cytokine family intracellular and extracellular processing. **(A)** *Intracellular processing*. Caspase-1 cleavage from the NLRP3 inflammasome activates pro-IL-1β and pro-IL-18 and inactivates pro-IL-37, which is translocated in the nucleus. IL-1α and IL-33 are rapidly released by damaged necrotic cells in their functionally active full-length precursors and are inactivated by caspase-1, 3, and 7 cleavage. Intracellular processing of IL-36α, IL-36β, IL-36γ, and IL-38 are still unknown. **(B)** *Extracellular processing*. Epithelial cells, NK cells, macrophages, mast cells, and PMN secrete proteases to activate IL-1β, IL-18, IL-33, IL-36α, IL-36β, and IL-36γ. Unlike the intracellular processing, the cleavage of IL-33 and IL-37 results in a superactive form. NK, natural killer; PMN, polymorphonuclear neutrophils; PR3, proteinase 3.

With the exception of IL-1α and IL-33, cytokine precursors of the IL-1 family generally require intracellular processing to generate active forms. However, extracellular processing also occurs, involving different proteases. Protease driven from PMN, including elastase and cathepsin G, as well as chymase from mast cells and macrophages, proteinase 3 from PMN and macrophages, granzyme B from natural killer (NK) cells, and meprims from epithelial cells, are able to convert pro-IL-1β, pro-IL-18, pro-IL-36 α, β, and γ into their corresponding fully active forms. Active pro-IL-33 can also be cleaved extracellularly by these proteases to generate a super active form (Figure 1).

### Immune Cell Targets of IL-1 Superfamily Members

#### IL-1 Superfamily Members and Myelopoiesis Control

While IL-1 superfamily members have a role in the activation and mobilization of monocytes and in the recruitment of PMN, they also fulfill a critical function in the induction of emergency hematopoiesis leading to sustained myeloid M1 skewing. In this setting, IL-1α and IL-1β are both liable to be produced by the medullar environment. However, emergency hematopoiesis and its pro-inflammatory component are strongly associated with IL-1β, which mainly acts systemically (see section Concept of Trained Immunity and Innate Immunological Memory: The Place of the IL-1 Cytokine Family: trained immunity), whereas IL-1α in its role as cytokine/alarmin seems to exercise juxtracrine action (9, 19–21).

#### Cytokines of the IL-1 Superfamily and Control of T Helper 1 Effectors and NK Cells

IL-18 and IL-1 are implicated in the genesis of type 1 responses through the recruitment and differentiation of T helper (Th)1 lymphocytes and the activation of NK cells and of innate lymphoid cells (ILC)1, particularly by inducing IFNγ production. In a mouse model, absence of IL-18 production or of its mature form leads to loss of anti-tumoral activity, loss that is partially objectified by the absence of the FasL-dependent cytotoxic function by hepatic NK cells, a circumstance favoring pulmonary and colorectal cancers (22).

The existence of antigen-specific and/or NK memory cells was recently described [for review (23)]. Remarkably, these are liver-resident NK cells serving as the support for memory responses in mice and, quite probably, in humans as well (23–25). That much said, in men as well as mice there exist several models suggesting that IL-18 is a factor implicated in the generation of antigen-specific and/or NK memory cells (26). However, study of mice genetically deficient in IL-18 or IL-18Rα shows that IL-18 is not essential in this function (24).

IL-1R8 seems to be an antagonist of IL-18 insofar as the effector/cytotoxic function of NK cells increase pronouncedly in mice genetically deficient in IL-1R8. Remarkably, these mice are resistant to the induction of hepatocellular carcinoma (HCC) or hepatic metastases of colorectal or pulmonary cancer. IL-1R8 at least partially exercises its antagonistic effects on the effector functions of NK cells by interfering with the IL-18/IL-18Rα/Myd88 signaling pathway. In humans, IL-37, of which the receptor consists in the IL-1R8 and IL-18Rα sub-units, is suppressive with regard to NK CD56(dim)CD57(+)FcεRγ(+)NKG2C(–) cells.

So it is that the cytokine/receptor couples IL-18/IL-1R5 and IL-37/IL-1R8 have opposed effects in the effector/cytotoxic functions of NK/cytotoxic cells.

According to organism site or the tissue, the effects of IL-1R8 favor or disfavor the emergence of infections and/or cancers. For example, IL-1R8 represents not only a protective factor as concerns the development of some colon cancers (11), infectious complications of keratitis (27), and acute *Pseudomonas aruginosa* pneumopathies (28), but also a promoting factor as concerns mammary cancer and HCC (10, 29). One interpretation of these opposed effects is that in the absence of IL-1R8, an exacerbated inflammatory response (bringing into play the different cell types sensitive to IL-18/IL-33 and IL-37) favors solid tumorigenesis and tissue lesions during infections, while censoring of cancers in liver, particularly hepatic metastases, is partially counteracted by IL-1R8.

IL-18BP (Binding Protein) is also involved and blocks IL-18 action and seems critical as a means of limiting deleterious hepatic NK responses in hepatic aggression models (30, 31) (see Acetaminophen-Induced ALF and Post-Viral Hepatitis A ALF).

#### IL-1α/β and Th17 Lymphocyte Differentiation

IL-1 superfamily members are cytokines of critical importance in control of IL-17 production and in differentiation of Th17 lymphocytes and ILC3 (9, 32, 33). This function of IL-1 superfamily members is synergic with that of IL-23. As a result, IL-1R1-deficient mice are lacking in differentiation of Th17 lymphocytes/ILC3 and protected from experimental autoimmune encephalitis induction (34, 35).

#### The Complex Effects of IL-33 on the Dynamics of Immune Response

- Pro-Th2 effects: IL-33 is a key factor in ILC2 and M2 macrophage differentiation/maturation and also intervenes in Th2 lymphocyte differentiation. From that standpoint, IL-33 has a critical role in the immune responses of mucus epitheliums, as has been demonstrated in mouse models of bronchial hyper-reactivity, pulmonary allergies, and pulmonary response to *A fumigatus* (13). Remarkably, IL-33 on ILC2 induces expression of amphiregulin (Areg), a factor implicated in tissue repair (36, 37).
- The facilitating effects of Th1/cytotoxic/NK cell response: In addition to its effects on type 2 immunity, IL-33 plays a role in control of T cytotoxic and NK cell responses. Associated with IL-12, IL-33 induces IFNγ production by NK cells and seems to have a spectrum of action similar to that of IL-18 (25, 38, 39). According to the timing of stimulation and its chronicity at the level of the bronchial mucus, IL-33 will either favor a type 2 response by means of ILC2 or, on the contrary, induce a pro-Th1 NK cell response, as in the case of chronic lung inflammation due to tobacco in chronic obstructive pneumopathy disease (40). In a context of antiviral response, IL-33 serves as an amplification factor of effector cytotoxic T CD8 cell response by favoring the expansion of short live effector cells (41). Lastly, IL-33, in combination with IL-12, induces IFNγ production by invariant natural killer T (iNKT) cells (38, 42). From this standpoint, we may note the role of the IL-33/iNKT/IFNγ axis in the genesis of renal ischemia-reperfusion (IR) injury in animal models and in humans following transplantation (43–45).
- A role in the recruitment and acquisition of T regulatory lymphocytes' innate competences IL-33 is implicated in the recruitment of T regulatory (Treg) lymphocytes during renal IR injury (46), and is associated with improved renal IR injury tolerance. Furthermore, the eutrophic functions of Treg lymphocytes present in visceral adipose tissue are dependent on the production of IL-33 by the stromal cells in adipose tissue (47, 48). Lastly, IL-33, and to a certain extent IL-18, are liable to induce Areg production independently of the T cell receptor (TCR) (innate Treg function), thereby contributing to restorative functions, particularly those of the epitheliums, and also favoring tumor promotion and growth (36, 49).

### Concept of Trained Immunity and Innate Immunological Memory: the Place of the IL-1 Cytokine Family

A series of recent studies has shown that non-specific stimulation of the immune system (pathogenic agent through *Mycobacterium tuberculosis* or BCG, oxidized low-density lipoproteins, Western diets…) induces modification of the pro-inflammatory immune response program driven by or dependent on myeloid cells (monocytes/macrophages) (19–21, 50, 51). Acquisition of this imprint depends on IL-1β. This inflammatory emergency hematopoiesis is implicated in the genesis of atherosclerosis lesions, metabolic syndrome, NAFLD or induction of tumor transformation, in particular by a promoting effect of epithelial-mesenchymal transition (EMT). It is the demonstration that this phenomenon leads to the constitution of an innate memory of cells of myeloid origin that led to the proposal of the new concept of “trained immunity.” We have attempted here to apply the concept of trained immunity to liver diseases, based on the role of the IL-1 superfamily, first of all IL-1β but also IL-18 and IL-33, in modulating innate lymphoid immunity carried by NK cells, ILC or innate T-αβ lymphocytes.

## Contribution of IL-1 Superfamily of Cytokines to Hepatic Diseases

### Constitutive and Inducible Expression of Members of the IL-1 Superfamily in the Liver

In steady-state, the liver expresses the IL-1α and IL-33 cytokines/alarmins, IL-18 cytokine, the receptors functioning as decoys or inhibitors of these three cytokines: IL-1Ra, IL-18BP, and IL-1R2. While pro-IL-1α is expressed in hepatocytes, pro-IL-18 is expressed in Kupffer cells (KC). IL-33 is constitutively expressed in liver vascular endothelial and liver sinusoidal endothelial cells (LSEC) (52). Such constitutive expression may have major implications during liver injuries in which cell death by necrosis is predominant and a rapid immune response is required.

Expression in steady-state in liver also occurs for anti-inflammatory factors such as IL-1Ra, which antagonizes IL-1-related functions and modulates a variety of immune and inflammatory responses (53) or IL-18BP, of which the spontaneous production neutralizes locally produced basal IL-18. The sources of IL-18BP in the liver seem to be the hepatocytes themselves, KC and hepatic stellate cells (HSC) (31). The soluble form of IL-1 receptor accessory protein (IL-1RAcP) is constitutively produced by the liver and forms a complex with the soluble IL-1RII, which binds and neutralizes IL-1β (8, 54). IL-33 and ST2 are also constitutively expressed in the normal liver (55).

Under pathological conditions that drive liver damage, IL-1 superfamily members are up-regulated. IL-1 and IL-1Ra/IL1-R1 are expressed in almost all cells, especially in activated non-parenchymal cells such as KC. As an alarmin, IL-33 is rapidly released from LSEC and vascular endothelial cells following carbon tetrachloride or Concanavalin A (ConA)-induced hepatitis (52) to mediate a pro-inflammatory response. In response to these danger signals, IL-33 may also be newly secreted in hepatocytes and/or HSC. Its specific receptor ST2 (IL-1 receptor-like 1) and co-receptor IL-1RAcP constitutively expressed on innate immune cells contribute to a rapid immune response.

### Acute Liver Failure

Acute liver failure (ALF) is defined as the rapid development (within days or weeks) of severe liver injury with impaired liver function and hepatic encephalopathy (56). ALF is a life-threatening condition and refers to a wide variety of causes, among which toxin/drug-induced (usually acetaminophen) liver damage or viral hepatitis (hepatitis A, B, and E) are most common in the United States and Europe (56, 57).

#### The Contributions of Experimental Models

*In vivo* studies in animals have shown the existence of mechanisms common to the different ALF models. So it is that cell death by necrosis results in rapid IL-1α precursor release, upregulation of IL-1β and IL-18 leading to tissue injury through the IL-1R/IL-18R-MyD88 pathway (58–60) with massive upregulation of anti-inflammatory molecule IL-1Ra (61). Following ALF, IL-1α, IL-1β, and IL-18 all up-regulate the pro-inflammatory process through a dramatic decrease in hepatic inhibitor of kappa B (IκB) levels and NF-κB pathway activation, leading to IL-6 and TNFα secretion, which contributes to apoptosis, and ultimately to liver damage and animal death (60).

##### Lipopolysaccharide-induced ALF

In the study by Tsutsui et al. (62) KC were shown to be the main producers of IL-18, acting not in a caspase-1-dependent manner but upon Fas Ligand stimulation, thereby increasing IFNγ and TNFα production, which causes substantial acute liver damage. Furthermore, Yan et al. demonstrated a correlation between IL-1 and MMP9 expression implicated in extracellular matrix degradation, sinusoidal collapse, leading to parenchymal cell death, and loss of liver function after ALF induction (63). Inhibition of the IL-1 pathway (using adenovirus IL-1Ra) before the induction of ALF led to a significant reduction in plasma levels of hepatic enzymes and to animal survival improvement (64). Lastly, in humans, treatment by recombinant human IL-1Ra (Anakinra) improved survival of patients with acute liver injury in a post-septic situation (65).

##### Acetaminophen-induced ALF

**Figure 2:**
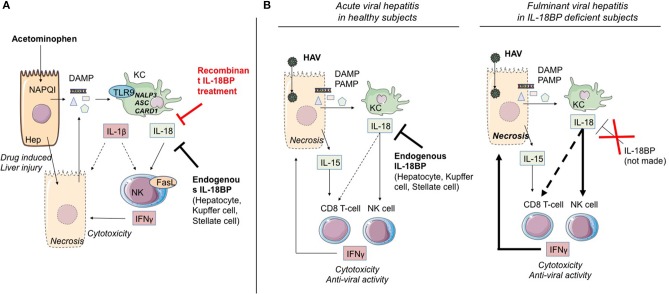
IL-1α/β and IL-18 members are involved in ALF. **(A)** *Acetaminophen-induced ALF*. NAPQI (N-acetyl-*p*-benzoquinone-imine) is a toxic acetaminophen metabolite generated by hepatic cytochrome Cype2e1 and Cyp1a2. Under NAPQI action, injured and necrotic centrilobular hepatocytes secrete DAMP. Among them, ATP and TLR9 receptor ligand induce IL-1β and IL-18 production by Kupffer cells. Both cytokines drive NK and iNKT cell killing function through FasL and IFN-γ production. This inflammatory process can be blocked either by endogenous IL-18BP production by non-damaged hepatocytes, Kupffer and stellate cells, or by recombinant IL-18BP treatment. **(B)** *Critical role of IL-18 in HAV fulminant hepatitis*. HAV-infected hepatocytes produce IL-15 as well as PAMP and DAMP which, in turn, activate IL-18 production by Kupffer cells. IL-18 activates IFN-γ production by NK cells and CD8 T-cells with innate functions, hence promoting cytotoxicity toward infected hepatocytes. In healthy infected subjects (left), endogenous IL-18BP blocks the deleterious IL-18 action and modulates NK cell and probably CD8 T-cell activation. In IL-18BP deficient subjects (right), there is no inhibition of the deleterious effects of NK cell and CD8 T-cell activation. ALF, acute liver failure; KC, Kupffer cells; NK, natural killer; Hep, hepatocyte; bounded arrow, presumably yes.

In the acetaminophen (acetyl-*p*-Aminophenol, APAP)-induced acute liver injury model, KC were shown to be the main producers of IL-1β and IL-18, in a TLR9-dependent induction and NALP3-ASC-caspase-1-dependent manner increasing/inducing IFN-γ and TNF-α production by Th1 and NK cells, which causes substantial acute liver damage (30, 62, 66) (Figure 2A). Overexpression of IL-18, known to induce FasL expression on NK cells and CD4(+) T cells, also increased expression of Fas on hepatocytes, which were thereby sensitized to NK cytotoxicity. These Fas/Fas Ligand interactions induced hepatocyte apoptosis, massive periportal fibrosis, inflammation, and severe liver failure (67). Remarkably, IL-18-deficient mice were resistant to ALF induction by APAP, and blocking of IL-1β by a neutralizing antibody reduced ALF severity (68). Moreover, using the same model, it was recently shown that treatment of mice by IL-18BP, which blocks the binding of IL-18 to its receptor, provided protection from induction of hepatic lesions, thereby confirming the critical role of IL-18/IL-18R-NK cells (30).

To further explore the mechanisms involving the IL-33/ST2 axis in ALF, Antunes et al. (69) used a mouse model of APAP-IL (-induced liver) failure and showed that liver necrosis was associated with massive IL-33 and chemokine release. Non-parenchymal liver cells were the main sensors of IL-33, and the inflammatory response triggered by IL-33 was amplified by liver PMN infiltration. In this model, IL-33 may have acted in synergy with IL-18 in the recruitment of NK cells.

IL-36 is induced after treatment with acetaminophen, presumably in hepatocytes, and up-regulates chemokine ligand 20, a chemokine implicated in tissue protection and repair. Administration of the antagonist IL-36Ra aggravates liver damage and disturbs tissue recovery (70).

##### Poly-I:C-induced ALF

In a mouse model of ALF induced by poly(I:C), IL-33 expression was up-regulated and correlated with severe liver injury. Interestingly, iNKT cell-deficient mice exhibited protection against poly(I:C)-induced hepatitis accompanied by an increased number of IL-33-positive hepatocytes compared with wild-type (WT) controls (52, 71).

##### Liver IR injury

Ischemia, followed by reperfusion at the time of liver graft implantation, leads to what is called IR injury. Understanding of the mechanisms involved could help to identify novel therapeutic targets in view of improving organ survival. In mice and rats, warm ischemia followed by reperfusion represents the most common model to study IR injury.

On the one hand, IL-1β and the NLRP3 inflammasome are implicated in the lesions of warm IR injury *via* high-mobility group box 1, NF-κB and toll-like receptor (TLR)4 (72). Reactive oxygen species (ROS) mediate inflammasome activation in KC (73). On the other hand, it has been suggested that NLRP3 could be involved in IR injury independently of inflammasomes through PMN recruitment (74). Sadatomo et al. (75) showed in a murine model that macrophages secreted pro-IL-1β, which is activated by neutrophils-derived proteinases. Interaction between neutrophils and macrophages promoted IL-1β maturation and causes IL-1β-driven inflammation in the IR liver.

Regarding the role of IL-33 and its receptor ST2 in warm IR injury, Yazdani et al. (76) demonstrated in a warm IR injury murine model (confirmed in human liver resection specimens) that IL-33 release from LSEC increases sterile inflammation and results in PMN extracellular trap formation, while administration of recombinant IL-33 during IR exacerbates hepatotoxicity and inflammation. One may note that iNKT cells mediate hepatic IR injury by promotion of intrahepatic PMN influx (77), similarly to what has been shown in models of IR injury involving other organs, such as the kidney (43). Interestingly, preconditioning with intra-peritoneal injection of IL-33 (55) showed that IL-33 has a protective effect on hepatocytes, with decreased liver IR injury *via* the activation of NF-κB, p38 MAPK, cyclinD1, and Bcl-2. Sakai et al. (78) used a mouse model of IR with injection of recombinant IL-37 at the time of reperfusion. They demonstrated that IL-37 protects against IR injury by reducing pro-inflammatory cytokine and chemokine production by hepatocytes and KC, and suppression of PMN activity.

#### Observational Clinical Studies

##### The pro-inflammatory/anti-inflammatory balance of IL-1 family members determines the clinical outcome

Clinical observations first revealed the contribution of the IL-1 family of cytokines to the pathophysiology of ALF. Significant increase in serum levels of the pro-inflammatory cytokine IL-1α (79), IL-1β (80), IL-18 and its activator Caspase-1 (81), IL-33 (82) positively correlates with acute hepatitis in humans. Both highly significant elevation in IL-1α levels (79) and reduction in the IL-1Ra over IL-1β ratio (IL-1Ra/IL-1β) (80) have been detected in ALF patients with a fatal outcome. These findings show that the IL-1 family members brought into play are systematically associated in the event of a fatal outcome with loss of balance in the liver between inflammatory signals IL-1α/β/IL-18 and anti-inflammatory IL-1Ra.

Upregulation of IL-33 and sST2 in serum has been shown to exist in ALF and acute-on-chronic liver failure in patients (82), correlating with the intensity of necrosis as assessed by transaminase activity. Interestingly, sST2 was elevated in acute and acute-on-chronic liver failure, but not in chronic liver failure, suggesting that sST2 could be a tool to monitor the course of the disease.

##### Post-viral hepatitis A ALF

Less than 1% of acute hepatitis A virus (HAV) infections result in acute fulminant hepatitis. A few cases of post-AHV ALF family isolates have been reported, suggesting the role of a genetic factor implicated in the determinism of this pathology. The study by Belkaya et al. (31) on an 11-year-old patient having died from fulminant HAV hepatitis, with no previous personal of familial medical history, identified a deletion of 40 nucleotides of the gene coding IL-18BP, leading to instability at the mRNA level, and loss of the normal structure, or the absence of IL-18BP.

During the acute phases of viral hepatitis, particularly HAV, massive secretion in patients' serum of IL-18 and IL-15 has been observed (83). Interestingly, the secretion is accompanied by the appearance of a cell population consisting in innate CD8 T lymphocytes (whose expansion and cytotoxic function are independent of TCR engagement) in the blood and the liver (83). It is highly likely that acquisition of this “innate memory” phenotype depends, as has been described above (section Cytokines of the IL-1 Superfamily and Control of T Helper 1 Effectors and NK Cells), on the joint action of IL-18 and IL-15. Belkaya et al. (31) went on to show that in the patient having succumbed to post-HAV ALF, the aforementioned absence of IL-18BP was accompanied by excessive activation of NK cells, activation that depended on IL-18. On the basis of their analysis of this clinical case, the authors hypothesized that the IL-18BP deficiency could partially explain the fulminant viral hepatitis (Figure 2B).

To sum up, the different findings from experimental models and from clinical situations involving ALF underline the importance of the implication of NK/T CD8 cells by means of IL-18 and IL-1 and confirm the decisive role of “negative” regulatory elements (IL-1R8, IL-18BP, IL-37) whose appearance attenuates or reduces the severity of acute hepatic inflammation (Figure 2).

### Alcoholic Liver Diseases

Alcoholic liver diseases (ALD) includes liver manifestations due to alcohol overconsumption: fatty liver, alcoholic hepatitis, and chronic hepatitis with liver fibrosis or cirrhosis.

**Figure 3:**
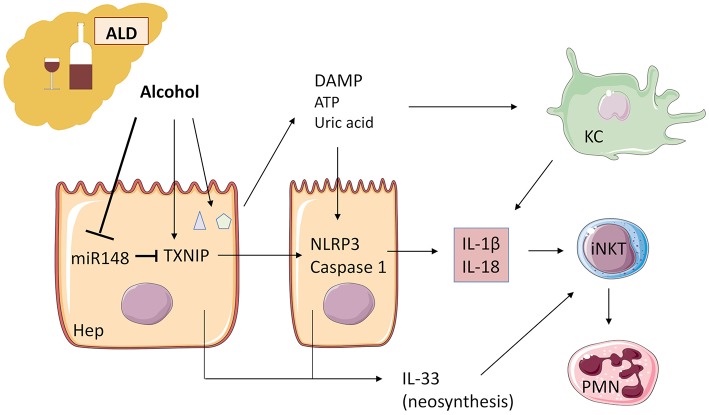
Alcohol acts as an “exogenous signal” on Kupffer cells to activate the inflammasome NLRP3-caspase 1 and production of IL-1β in ALD. Alcohol decreases microRNA miR-148a, a negative regulator of TXNIP (thioredoxin-interacting protein), which is overexpressed during ALD. TXNIP activates NLRP3 inflammasome in hepatocytes and caspase 1-mediated pyroptosis, leading to IL-1β and IL-18 production. ATP and uric acid release by damaged hepatocytes after alcohol damage is a second “endogenous signal” leading directly and/or through Kupffer cell activation to inflammatory cytokine production. IL-1β and IL-18 recruit and activate iNKT cells that, in turn, induce PMN afflux and hepatic injury. IL-33 released by damaged and necrotic hepatocytes exacerbates iNKT cells and PMN recruitment and activation. ALD, alcoholic liver disease; KC, Kupffer cells; iNKT, invariant natural killer T-cells; PMN, polymorphonuclear neutrophils; Hep, hepatocyte.

Alcohol acts as an “exogenous signal” on KC through TLR with activation of the inflammasome NLRP3-caspase 1 and production of IL-1β. A second “endogenous signal” leading to activation of the same pathway, is the release of two DAMP: ATP and uric acid by damaged hepatocytes because of alcohol damage (84). Upregulation of IL-1β activity leads to inflammation, steatosis, and additional damage (85, 86) (Figure 3).

Recently, Heo et al. (87) demonstrated in human and murine liver samples that alcohol decreases microRNA (miR)-148a through the transcriptional regulator forkhead box protein O1. TXNIP (thioredoxin-interacting protein) is a direct target of miR-148a and is overexpressed during alcoholic liver disease, activating NLRP3 inflammasome in hepatocytes, and caspase 1-mediated pyroptosis. Hepatocyte-specific delivery of miR-148a to mice abrogates alcohol-induced TXNIP overexpression and inflammasome activation (Figure 3).

In human patients with alcoholic liver disease, sST2 but not IL-33 was correlated to the severity of the disease (88). In the same fashion, Wang et al. (89) showed with IL-33- and ST2- deficient mice that ST2 decreases the inflammatory activation of hepatic macrophages by inhibiting NF-κB in alcoholic liver disease, in an IL-33-independent manner. However, during severe liver injury, massive cell death, and release of IL-33 triggers IL33/ST2 signaling and increases tissue damage, presumably by local activation of different cells expressing ST2, namely hepatic NK cells, iNKT cells, ILC2, and Treg lymphocytes.

During ALD, one target of IL-1β, and possibly IL-33, is the iNKT cell population (86). From this standpoint, it is interesting to note that mice genetically deficient in Jα18 T cells or CD1d, and consequently without iNKT lymphocytes, are at least partially protected from ALD (90, 91). In these models, iNKT lymphocytes are responsible for PMN influx during ALD progression.

### Non-alcoholic Fatty Liver Disease

With prevalence of 30% for adults and 10% for children in Western countries, non-alcoholic fatty liver disease (NAFLD) is a growing public health issue frequently associated with morbid obesity, type 2 diabetes, and metabolic syndrome (92, 93). NAFLD is characterized by excessive fat accumulation in the liver associated with insulin resistance, and is defined by the presence of steatosis in hepatocytes. NAFLD ranges from simple steatosis or non-alcoholic fatty liver to a more serious form called non-alcoholic steatohepatitis, which is characterized by liver inflammation and tissue damage that can lead to fibrosis, cirrhosis, and hepatocellular carcinoma (94).

**Figure 4:**
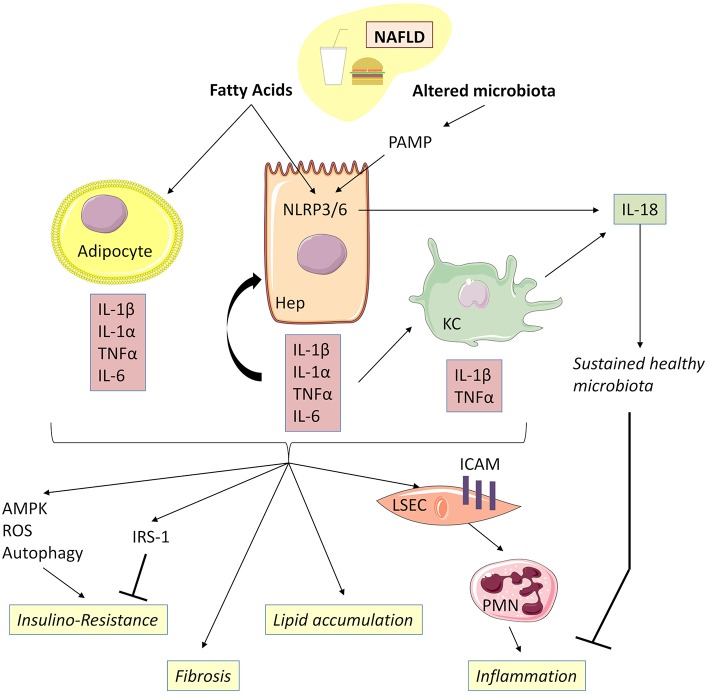
IL-1β, together with TNFα, mediates low-grade inflammation during NAFLD. Free fatty acids, acting as DAMP, are capable together with TLR ligands of activating NLRP3 and NLRP6 inflammasomes, thereby inducing caspase-1 activation and release of IL-1α and IL-1β in hepatocytes and Kupffer cells. Adipocytes could be an important source of IL-1α and IL-β during NAFLD associated with obesity. Adipocyte-derived IL-1β has been shown to directly induce insulin resistance in hepatocyte development of insulin resistance in adipocytes enabling accumulation of lipids in the liver. IL-1β is also capable of reducing insulin receptor substrate-1 (IRS-1) expression dependently and independently of ERK activation and of inducing impairment in insulin signaling and action. IL-1β is released by hepatocytes through inflammasome activation and, in turn, amplifies inflammasome activation, TNFα, and IL-β release in Kupffer cells. Increased IL-1β and NLRP3 drive liver fibrosis in experimental models of NAFLD mice. IL-1β acts on LSEC to promote liver inflammation by upregulating ICAM-1 (intercellular adhesion molecule 1) expression, which stimulates neutrophil recruitment in the liver. IL-18 production negatively regulates NAFLD/NASH progression *via* modulation of the gut microbiota. NAFLD, Non-alcoholic fatty liver disease; KC, Kupffer cells; LSEC, liver sinusoidal endothelial cells.

There is increasing evidence that the induction of inflammation and production of inflammatory mediators released from the adipose tissue of obese subjects, such as adipocytokines and classical cytokines contribute to obesity-induced NAFLD, in which IL-1α/β plays a key role (Figure 4) (95, 96). In fact, NAFLD is the last step of low-grade inflammation depending on the bringing into play of “trained immunity”.

Free fatty acids, acting as DAMP, are capable together with TLR ligands present in gut microbiota of activating NLRP3 and NLRP6 inflammasomes, thereby inducing caspase-1 activation and the release of IL-1α, IL-1β, and IL-18 in hepatocytes (97, 98). IL-1α/β, in turn, activates inflammasome, TNFα and IL1β and IL-18 release in KC (98). Other extra-hepatic cell types such as adipocytes could also be important sources of IL-1α and IL-1β during NAFLD associated with obesity (99).

IL-1β promotes hepatic steatosis by stimulating triglyceride and cholesterol accumulation in primary liver hepatocytes and lipid droplet formation (96) and acts on LSEC to promote liver inflammation by upregulating intercellular adhesion molecule 1 expression, which stimulates neutrophil recruitment in the liver (100). Moreover, IL-1β produced by the liver, together with pro-inflammatory IL-6 and TNFα, contributes to the activation of resident immune cells and the recruitment of other leucocytes to the damaged liver, leading to chronic inflammation (101). Accordingly, IL-1β and/or IL-1α-deficient mice have demonstrated less diet-induced inflammation and liver fibrosis, as attested by lower serum transaminases and serum amyloid alpha concentrations, as well as decreased expression of inflammatory and fibrosis transcripts such as *IL-6, TNF*α, *P-Selectin, Cxcl1*, and *TGF*β, as compared to WT controls (102).

IL-1α/β are upregulated in the adipose tissue of obese and insulin-resistant mice. Together with TNFα and IL-6, IL-1α/β, in turn, contributes to the development of insulin resistance in adipocytes through downregulation of insulin receptor substrate-1 expression, thereby facilitating accumulation of lipids in the liver (103). Moreover, adipocyte-derived IL-1β has been shown to directly induce, alone or in combination with TNFα, insulin resistance in hepatocytes *via* AMPK-ROS signaling and autophagy (104, 105). Accordingly, IL-1α and IL-1β -deficient mice have lower fasting glucose and insulin levels and improved insulin sensitivity (106).

Of note, IL-1α deficiency increased both hepatic and systemic cholesterol levels, suggesting that liver fat storage and inflammation are not necessarily parallel events and that some hepatic lipids might support anti-inflammatory functions (102). IL-1Ra-deficient mice exhibited severe steatosis and pericellular fibrosis containing many inflammatory cells, as observed in steatohepatitis histological lesions in humans, following 20 weeks of feeding with an atherogenic diet (107). These different observations, particularly as regards the role of IL-1Ra in NAFLD, raise the question of the interest of therapeutic utilization of human recombinant IL-1Ra (Anakinra) in the natural history of NAFLD.

Henao-Mejia et al. (108) have reported that changes of gut microbiota linked to NLRP3 and NLRP6 inflammasome deficiency were associated with increased hepatic steatosis and inflammation. Interestingly, IL-18 production negatively regulates non-alcoholic steatohepatitis progression *via* modulation of the gut microbiota. This observation strongly highlights the role of the maintenance of digestive microbiome homeostasis in the genesis of “metabolic syndrome” and NAFLD (108).

Regarding new members of the IL-1 superfamily, IL-33 has not been widely explored. In a murine model, a high fat diet (HFD)-induced steatohepatitis was associated with upregulation of IL-33 but IL-33 deficiency did not affect severity of liver inflammation or liver fibrosis, suggesting that endogenous IL-33 has no effect on the progression of fibrosis during experimental steatohepatitis (109). Treatment with recombinant IL-33 in mice attenuated diet-induced hepatic steatosis but aggravated hepatic fibrosis in a ST2-dependent manner (110). The pro-fibrotic effect of pharmacological IL-33 treatment in HFD mice was confirmed in another study (111), where galectin-3 enabled upregulation of the IL-33/ST2 pathway and production of IL-13 by peritoneal macrophages.

### Autoimmune Hepatitis

**Figure 5:**
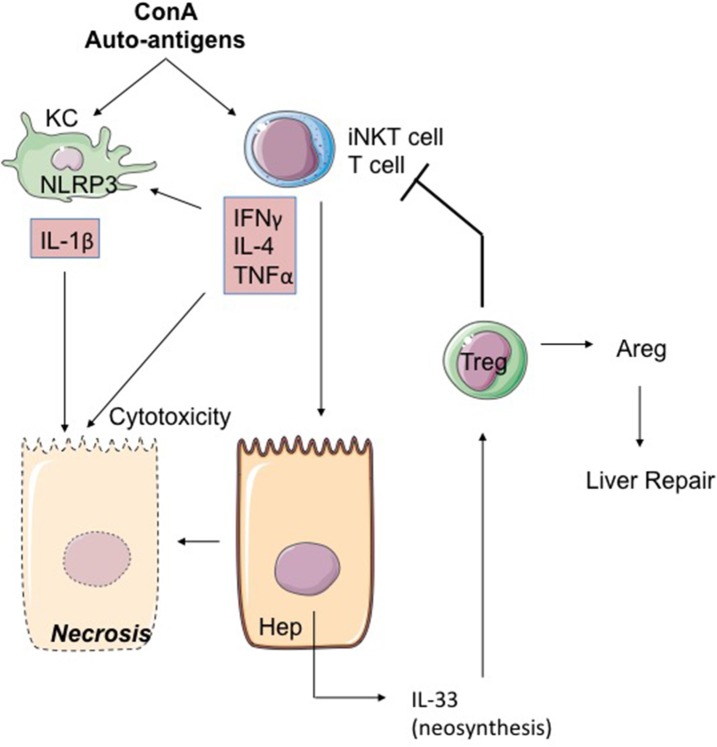
The ConA model of hepatitis. The mitogen Concanavalin A (ConA) activates iNKT cells in a KC and iNKT cell-dependent mode. IL-4, IFNγ, and TNFα produced by hepatic activated iNKT cells activate the NLRP3 inflammasome leading to toxic IL-1β production by hepatocytes. iNKT cells could also induce IL-33 production by hepatocytes through TRAIL/DR5 interactions. Secreted IL-33 acts as a retro-negative regulator of iNKT cells by recruiting Treg. Indeed, Treg can restrain T-cell cytotoxicity and promote repair through signals like Areg. ConA, concanavalin A; Areg, Amphiregulin; KC, Kupffer cell; Hep, hepatocyte.

Autoimmune hepatitis (AIH) is a chronic inflammatory disease of the liver of which the pathogenic mechanisms have yet to be unraveled. Treatment relies mainly on lifelong immunosuppressive therapy and liver transplantation in the final stage. Acute hepatitis induced with ConA is the most widely used mouse model of AIH (Figure 5). In this model, massive activation of immune cells induces massive local production of cytokines such as IL-4, IFNγ, and TNFα (112–114), which are directly implicated in hepatocyte death (113). iNKT lymphocytes are of major importance in acute hepatitis induced with Con-A, as is attested in works showing that iNKT lymphocyte-deficient mice resist hepatitis induction (115). Aside from their cytokine production, the pathogenic effect of iNKT lymphocytes depends on their cytotoxic functions, which bring into play perforin and granzymes as well as FasL/Fas and TRAIL/DR5 interactions (115, 116). Depletion of KC prior to ConA treatment attenuates hepatic injury (117). Activation of iNKT lymphocytes may consequently depend on KC, thereby ensuring a role of auto-antigen presentation. Another pathogenic mechanism may consist in activation of KC by iNKT lymphocytes, leading to production by the KC of TNFα, ROS and IL-1β depending on the activation by ROS of the NLRP3 inflammasome (118). On this subject, it is interesting to note that in this model, treatment of mice by human recombinant IL-1Ra (Anakinra) at least partially prevents hepatic lesions (118).

IL-33 is rapidly neosynthesized (6 hours) in the hepatocytes after treatment by ConA (116, 119) (Figure 5). Interestingly, this expression of IL-33 depends on the presence of iNKT lymphocytes and possibly on TRAIL/DR5 interactions between iNKT lymphocytes and hepatocytes (116). IL-33 production seems to moderate hepatitis severity, because: (i) IL-33-deficient mice have more heavily damaged livers than WT mice (52, 71, 116, 119, 120); (ii) injection of recombinant IL-33 renders less severe the hepatitis induced by ConA (120). Biologically, the increased severity of hepatitis lesions in IL-33-deficient mice is associated with pronounced expression of TNFα and IL-1β and is accompanied by accumulation in the liver of activated NK cells (119).

The protective action of IL-33 in the model of ConA-induced hepatitis may be attributed to the induction of anti-apoptotic factors such as BAX or Bcl2 (120). Another element associated with the protective action of IL-33 may be the recruitment in the liver of Treg lymphocytes [TCR-αβ(+)CD4(+)CD25(+)FoxP3(+)] ST2(+) (119). One is tempted to suggest that these Treg ST2(+) cells contribute to hepatic repair by expression of trophic factors such as Areg, as previously described in other tissues (36, 121).

### Viral Hepatitis B, C

According to the World Health Organization, 325,000,000 persons are currently living with a chronic infection of either hepatitis virus B (HBV) or hepatitis virus C (HCV). Infection with HBV rarely leads to acute liver failure. Chronic infections with both HBV and HCV are responsible for progressive fibrosis and cirrhosis, as well as development of hepatocellular carcinoma.

IL-1β and IL-18 may play a role in liver inflammation in the setting of HCV infection. Monocyte-derived human macrophages and Kupffer cells (122) produce IL-1β and IL-18 in response to HCV infection: HCV induces pro-IL-1β and pro-IL-18 in macrophages *via* the NF-κB signaling pathway. It has been shown that viral RNA can trigger MyD88/TLR7 pathway, thereby inducing IL-1β expression. HCV concomitantly activates the NLRP3 inflammasome, leading to IL-1β secretion (123).

It has been shown in humans that with their IFNγ production, iNKT lymphocytes inhibit the replication of HCV in hepatocytes (124) and that over the course of HBV infection, recruitment of iNKT lymphocytes occurs at the level of the liver (125–127). Lastly, in a model of HBV-transgenic mice, the authors showed that IL-18 induces repression of viral replication in a context depending on local recruitment of NK cells and iNKT lymphocytes (128). Interestingly, when infection becomes chronic, hepatic iNKT lymphocytes are progressively reduced in number (127), conceivably resulting in a loss of sensitivity of iNKT lymphocytes to IL-18 and/or IL-33.

From this standpoint, IL-33 expression in LSEC significantly increases in patients with chronic HBV (CHB) infection, and is likewise highlighted in the hepatocytes of lesions/inflammatory foci in patients with the most severe form of hepatitis (129). Seric IL-33 and ST2s concentrations are highly elevated compared to those in healthy donors and, interestingly enough, they are correlated with the severity of hepatic cytolysis (129, 130). In HBV acute-on-chronic liver failure (ACLF) (129), seric IL-33 and ST2s concentrations are pronouncedly higher than the concentrations observed in CHB patients. The circulating monocytes in ACLF patients present a highly activated and pro-inflammatory phenotype, which is correlated with IL-33 production. For that reason, it has been suggested that IL-33 increases inflammation and disease severity through monocyte activation, TNFα, IL-6, and IL-1β secretion. Similarly, IL-33 levels in human patients correlate with HCV RNA and liver damage (131). Such IL-33 production, particularly in the early phases of infection, can act in synergy with IL-18 and IL-1α/β in the recruitment of NK, iNKT, and T cytotoxic cells. And given the chronicity of its production, IL-33 is also liable to induce a state of systemic inflammation and, locally, a phenomenon of immuno-subversion through recruitment of Treg lymphocytes and ILC2 as opposed to recruitment and activation of NK and iNKT cells.

### Fibrosis

Liver fibrosis, the final stage of most types of chronic liver diseases, characterized by the excessive accumulation of extracellular matrix proteins, including collagen, is a consequence of extracellular matrix hepatic stem cell activation. Gieling et al. (132) established that IL-1 activates hepatic stem cells that produce MMP-9 and control the progression from liver injury to fibrogenesis. Advanced liver fibrosis results in cirrhosis with progressive liver failure and development of portal hypertension.

Marvie et al. (133) showed that in chronic hepatitis, in the model induced by CCl_4_, IL-33 and ST2 expression correlates with fibrosis (133). In humans suffering from chronic hepatitis, there also exists at the level of the liver a correlation between IL-33 cellular expression and fibrosis severity (133). In models of fibrosis induced by CCl_4_, thioacetamide, and bile duct ligation, increased hepatic IL-33 expression in activated HSC has been confirmed. Remarkably, fibrosis lesions are absent or non-severe in IL-33-deficient mice, thereby underscoring the major role of IL-33 in the manifestations of fibrosis (134). Lastly, this study suggests that the profibrosis function of IL-33 depends on its action in the activation and expansion of liver ILC2 (134). In the absence of IL-13 produced by ILC2, CCl_4_ does not induce hepatic fibrosis.

The fibrosis lesions described in the aforementioned works depend on the impact of environmental, chemical or physical stress. It may be the case that in the induction of hepatic fibrosis coming about during NAFLD, IL-33 is non-indispensable. This hypothesis is premised on the fact that in a model of hepatic fibrosis induced by HFD, there is no difference in fibrosis severity between WT and genetically IL-33-deficient mice (109).

### Hepatocellular Carcinoma (See Figure 6)

As described in section Cytokines of the IL-1 Superfamily and Control of T Helper 1 Effectors and NK Cells, the IL-18/IL-18R axis is a checkpoint driven by the immunological components controlling carcinogenesis and hepatic metastases. In addition to NK lymphocytes, iNKT lymphocytes are of key importance in the censoring of hepatic metastases (135, 136). As a result, iNKT lymphocytes strongly express IL-18 and IL-33 receptors (38, 39, 137) and may consequently be implicated in the anti-tumoral effect of the IL-18/IL-18R axis. Moreover, influx in the liver of iNKT lymphocytes depends on the expression of CXCL16 by LSEC, that is to say cells of which another characteristic is nuclear expression of IL-33. The study by Ma et al. (135) shows that recruitment of anti-tumoral iNKT lymphocytes in the liver is indirectly controlled by the digestive microbiota and by the metabolites produced by the latter, metabolites that will induce CXCL16 expression by LSEC. From this standpoint, it should be pointed out that there exists an accumulation of iNKT CD4(+) cells with a type Th2 profile in HCC liver (138). Enriched with iNKT cells, its infiltration is correlated with HCC progression, attesting to the loss of effectiveness of the anti-tumoral activity of iNKT lymphocytes in a tumor niche during the natural history of HCC.

**Figure 6:**
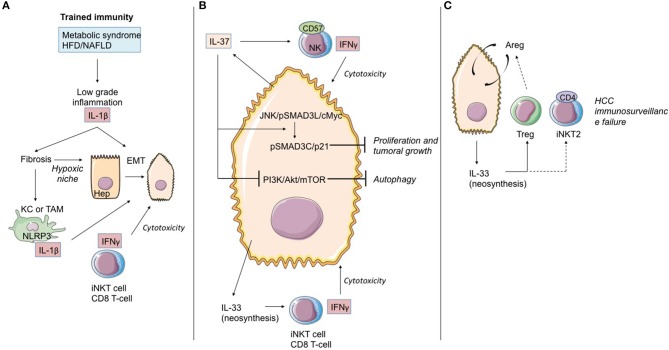
Role of IL-1 cytokine family members in the immunological history of the hepatocarcinoma. **(A)** *IL-1*β *cytokine and trained immunity in initiation of hepatocarcinoma transformation*. Trained immunity induced by metabolic syndrome or HFD/NAFLD leads to low-grade inflammation linked to IL-1β and other cytokine (as TNF-α) production. These cytokines induce hepatic fibrosis, exposing hepatocytes to moderate hypoxia. HIF-1α accumulation up-regulates IL-1β secretion in TAM/KC. Locally produced IL-1β induces or enhances epithelial-mesenchymal transition in cancer cells. At that time, iNKT cells and CD8 T-cells maintain the immunosurveillance of the HCC. **(B)** *IL-37 driven-suppression of hepatocellular carcinoma*. Hepatocytes and transformed hepatocytes produce IL-37, a cytokine with several cell targets. IL-37 suppresses hepatocellular carcinoma growth by converting pSmad3 signaling from JNK/pSmad3L/c-Myc oncogenic signaling into pSmad3C/P21 tumor-suppressive signaling. IL-37 inhibits the PI3K/AKT/mTOR pathway, inducing autophagy in hepatocellular carcinoma cells. IL-37 recruits cytotoxic anti-tumoral NK cells harboring the terminal differentiation marker CD57. At that time, IL-33 neosynthesis by hepatocarcinoma cells and hepatocytes could maintain the immunosurveillance by maintaining iNKT1 cell and T CD8 T-cell activation. **(C)** *Immunosurveillance failure at the end-stage HCC*. Sustained IL-33 production by tumoral cells can recruit Treg lymphocytes and promote development of iNKT2 CD4(+) cells, both leading to anti-tumoral immunosurveillance failure. Areg, Amphiregulin; EMT, epithelial-mesenchymal transition; HCC, hepatocellular carcinoma; KC, Kupffer cells; TAM, tumor-associated macrophages; bounded arrows, presumably yes.

Several works in mouse models have shown that endogenous IL-33 is an adjuvant factor in anti-tumoral and anti-metastatic immunity, including at the level of the liver (139–141). The limitation of these studies resides in the utilization of metastasis models, some of which use HCC cell lines but do not reproduce the natural history of HCC.

Even though HCC may develop in healthy liver parenchyma, in most cases it develops in chronic inflammatory liver parenchyma in pathology secondary to the induction of trained immunity depending on IL-1β production (NAFLFD, ALD, Viral chronic hepatitis, hepatic fibrosis). As we previously discussed, the liver possesses its own means of induction and control of oncogenesis. Consequently, the process of transformation/oncogenesis at the hepatic level is partially dependent on IL-1β and will avail itself of amplification paths.

Recently, Zhang et al. (142) demonstrated in cell cultures, mouse models, and human samples the existence of a hypoxia-inducible factor (HIF)-1α/IL-1β signaling loop between cancer cells and tumor-associated macrophages (TAM) in a hypoxic microenvironment, resulting in cancer cell epithelial-mesenchymal transition and metastasis. When exposed to moderate hypoxia, HIF-1α accumulation up-regulates IL-1β secretion in TAM. IL-1β increases inflammatory signaling and enhances epithelial-mesenchymal transition in cancer cells through the IL-1β/HIF-1α/COX2 axis, which promotes the invasive capacities of tumoral cells in a hypoxic microenvironment.

IL-37 is an anti-inflammatory cytokine, which in numerous models seems to block the deleterious effects of IL-1α/β and IL-18. Schematically, this cytokine counteracts the induction of trained immunity. From this standpoint, it is altogether remarkable that hepatic expression of IL-37 decreases during HCC evolution and that it does so both in the tumor itself and the non-tumoral tissue adjacent to the tumor. Factors suggesting that IL-37 may possess an anti-tumoral effect are corroborated by the following points: (i) persistence of hepatic expression of IL-37 is associated during HCC with infiltration of NK CD57(+) cells. *In vitro*, the HCC cell lines expressing IL-37 attract NK CD57(+) cells which, in turn, are cytotoxic with regard to the HCC lines (143); (ii) the profusion of NK CD57(+) cells and the elevated level of hepatic expression of IL-37 are associated with improved overall survival (143); (iii) IL-37 has an anti-tumoral effect during HCC. While the JNK/pSmad3L/c-Myc pathway is associated with tumor growth during HCC, the signaling depending on IL-37 diverts pSmad3L from that platform and associates it with the suppressor of tumor p21 in a pSmad3C/p21 complex. The presence of pSmad3L is inversely correlated with overall survival (144); (iv) IL-37 is liable to induce autophagia in the HCC cell lines by inhibiting the PI3K/Akt/mTOR signaling pathway, thereby sensitizing HCC cells to apoptosis induction (145).

As regards IL-33, data from the natural history of HCC are available in humans. Yang et al. (146) detected IL-33 protein expression by immunohistochemistry in patients with HCC, liver cirrhosis, hepatitis, and normal livers. They showed that expression of IL-33 is increased in HCC and LC patients, as compared to healthy donors. In HCC patients, IL-33 is expressed by both tumoral cells and peri-tumoral tissue. Immunohistology results in this work are corroborated by quantitative RT-PCR data. A second study (147) likewise underlines IL-33 expression at the level of the tumor during HCC. IL-33 seric expression is particularly pronounced when the disease has evolved, whereas high seric concentrations of ST2s have been found to be associated with reduced overall survival of patients with HCC (148).

High expression of IL-33 could be a factor contributing to deregulation of local response, possibly increased by desensitization of iNKT and NK lymphocytes as well as the recruitment of Treg lymphocytes and ILC2 producers of AReg, a cytokine of which the function in transformation and progression of HCC has been documented (149).

## Therapeutic Perspectives

Although the role of some members of the IL-1 cytokine family in liver disease has been extensively studied (IL-1α, IL-1β, IL-33), leading to strong arguments favoring these molecules as potential therapeutic targets, the role of the other members (IL-36, IL-37, IL-38) remains to be elucidated. Furthermore, some members of the IL-1 family are pro-inflammatory and enhance tissue damage and inflammation, while other members are more protective/anti-inflammatory by promoting tissue regeneration and preventing tissue damage and inflammation. Hence, these cytokines represent crucial targets for liver disease therapies and could open new perspectives of potentially innovative therapeutic approaches aimed at controlling local immune response and at limiting liver injury.

Members of the IL-1 superfamily have multiple cellular sources and targets, as well as numerous natural inducers and inhibitors. The pharmacological agents that can either suppress cytokine production or block their biological actions may have potential therapeutic value against a wide variety of liver diseases.

Moreover, several members of the IL-1 cytokine family can activate (or inhibit) the same receptors. For example, IL-1RAcP is shared by IL-1α, IL-1β, IL33, and IL-36, highlighting redundancy and compensatory actions. Since IL-1α, IL-1β, IL-33, and IL-36 are implicated *in vivo* during liver diseases, the efficiency of the therapeutic approaches based on targeting a cytokine by the other members may be affected. In other words, when targeting the shared receptors, in this case IL-1RAcP may be more effective. In addition to redundancy, some cytokines in the IL-1 superfamily are known for their duality of function. For example, IL-33 has shown both pathological and hepatoprotective functions and rIL-33-ST2 therapeutic possibilities seem to depend on the presence of different inflammatory conditions, which may require either activation or inhibition of this pathway.

### Modulation of IL-1β and IL-18 by Interference With Inflammasome

While some pharmacological agents against the NLRP3–caspase-1 pathway have been developed, their efficiency regarding cytokine inhibition and liver inflammation is moderate (106, 150, 151). This may be explained by the implication of additional mechanisms of extracellular activation of the cytokines. In addition to the classical NLRP3 inflammasome–caspase-1 cytokine activation pathway, serine proteases from immune cells (Figure 1) are also potent cytokine activators contributing to liver disease progression. The use of agents acting against those serine proteases, combined or not with anti-inflammasome-caspase-1 therapy, can be considered as a valuable therapeutic strategy and merits further investigation (152).

### Manipulation of the IL-1/IL-1R Pathway in Chronic Inflammatory Hepatopathies

IL-1β is a critical mediator of trained immunity and chronic inflammatory hepatopathies such as NASH and NAFLD, raising the question of the therapeutic targeting of the IL-1/IL-1R axis.

Anakinra is the recombinant form of IL-Ra used in treatment of chronic or acute inflammatory pathologies (Muckle-Wells, adult-onset Still's disease). It effectively treats macrophage activation syndrome (MAS), as regards both MAS associated with inflammatory pathologies such as Still's disease and MAS associated with septic shock (for a review, see 5, 63). Altogether remarkably, in the latter indication, Anakinra clearly improves survival of patients with disseminated intravascular coagulation or hepatobiliary injury (65).

Several studies have suggested that utilization of Anakinra in precancerous states slows evolution of the disease, particularly in cases of smoldering myeloma (associated with Dexametasone) (153). Moreover, it is a treatment that seems beneficial in management of some cancers, particularly metastatic colorectal cancer (154). It is also remarkably well-tolerated, with relatively few adverse effects, especially infectious [for a review, see (8)].

Blockage of the IL-1/IL-1R pathway can also be achieved with a neutralizing antibody directed against IL-1β, Canakinumab, which seems beneficial in atherosclerosis prevention in high-risk patients (155).

Most of these diseases involve inflammatory pathways depending on the bringing into play of trained immunity, of which we previously noted the role/implication in the genesis of different hepatopathies. While blockage of the IL-1/IL-R pathway is a therapeutic option that may be envisioned in cases of chronic inflammatory hepatopathies, conclusive demonstration of its effectiveness has yet to be provided.

### IL-18BP in ALF and Acute Viral Hepatitis

Recent demonstration of the role of the IL-18/NK cell axis in ALF genesis, particularly in cases of viral origin (31), has underscored the critical role of negative regulatory signals characterizing the pathway: IL-1R8 and IL-18BP. From this standpoint, in terms of therapeutic manipulation, IL-18BP is an excellent candidate. A human recombinant form (Tradekinig Alfa, AB2 Bioltd) is currently under evaluation in treatment of Still's disease and hemophagocytic lymphohistiocytosis syndrome, that is to say a pathology in which the IL-18/IL-18R axis seems preponderant. It has also been suggested that IL-18BP can prevent acetaminophen-mediated hepatotoxicity (30, 68). These different studies raise the issue of pharmacological targeting of IL-18 in a wide range of ALF cases.

### Recombinant IL-233

In situations of acute suffering due to IR injury, targeting of the IL-33/ST2 pathway is an option of genuine interest that nonetheless raises questions of intervention timing. Its application in organ preconditioning could represent a protective strategy, given that during an IR injury phase IL-33 is likely to be implicated in the appearance of lesions.

Another strategy aimed at favoring the recruitment of Treg lymphocytes during an IR injury sequence consists in the utilization of recombinant IL-233. This chimeric IL associates IL-2 and IL-33 functions: the N-terminal residues 21–169 of the IL-2 receptor are linked by 15 amino-acyl residues to C-terminal residues 109–266 of the IL-33 receptor (156). Recombinant IL-233 enables preferential recruitment of T lymphocytes and ILC2 at the expense of iNKT lymphocytes, NK cells and ILC1. Stremska et al. (156) have shown that in a renal IR injury model, recombinant IL-233 is “formidably effective” as a means of protecting mice at post-sequence IR injury. In addition to a modified balance of recruitment between pro-inflammatory effectors (iNKT/T/NK/PMN) and anti-inflammatory effectors (Treg lymphocytes/ILC2), it is altogether probable that in this model, IL-233 promotes rapid, and effective repair signals. It is consequently relevant to ask whether this molecule may be beneficially utilized in cases of acute hepatic inflammation, especially those associated with liver transplantation.

Even though it currently remains speculative, future therapeutic utilization of recombinant IL-233 in cases of chronic inflammatory hepatopathies merits consideration. A recent study highlighted the beneficial effects of IL-233 in prevention of diabetic nephropathy in an Ob type 2 diabetes (DT2) mouse model. In this mouse model of predisposition to obesity and DT2 on account of an Ob mutation of leptin rendering the latter non-functional, IL233 treatment during or after the appearance of DT2 provides protection from diabetic nephropathy (46).

These results give rise to the question of the possible interest of a “humanized” form of this molecule in the prevention of long-term consequences of trained immunity, especially as regards the liver. However, other questions arise: Might there not ensue an effect stemming from loss of cancer censuring and, eventually, promotion of tumor growth depending on the Treg/ILC2 lymphocytes expressing Areg?

### Conclusion Concerning Therapeutic Manipulation of Cytokines in the IL-1 Family

Further *in vivo* studies are needed to understand the potential benefits of targeting these shared receptors or simultaneously blocking multiple members of the IL-1 cytokine family.

Furthermore, these cytokines require enzymatic or proteolytic processing to become active, and their activators contribute to the process of liver inflammation and can therefore be potential therapeutic targets.

In conclusion, acute and chronic inflammations of the liver have various etiologies, ranging from toxic aggression (alcohol, fat diet, drugs) to viruses. Chronic liver inflammation leads to fibrosis and end-stage cirrhosis, and predisposes to the development of neoplasms such as hepatocellular carcinoma. The different members of the IL-1 superfamily of cytokines, which contribute to control of tissue homeostasis and help the liver to respond to damage and disease by promoting immune responses, have a major role in most liver inflammatory diseases and may represent potential clinical targets.

## Author Contributions

AR, CS, and ES contributed to literature search and editing of the review. LB, MF, AB, J-MG, and AH contributed to literature search for the review and provided writing and editing of the review.

### Conflict of Interest Statement

The authors declare that the research was conducted in the absence of any commercial or financial relationships that could be construed as a potential conflict of interest.

## Abbreviations

AID: induced cytidine deaminase
ACLF: HBV acute-on-chronic liver failure
ALD: alcoholic liver disease
ALF: acute liver failure
Areg: Amphiregulin
BP: binding protein
CHB: chronic HBV
ConA: concanavalin A
DAMP: damage-associated molecular pattern
DT2: type 2 diabetes
EMT: epithelial-mesenchymal transition
ERK: extracellular signal-regulated kinases
HAV: hepatitis virus A
HBV: hepatitis virus B
HCV: hepatitis virus C
HCC: hepatocellular carcinoma
HFD: high fat diet
HIF: hypoxia-inducible factor
ICAM-1: intercellular adhesion molecule 1
IκB: inhibitor of kappa B
IL: interleukin
IL-1Ra: IL-1 receptor antagonist
IL-1R: IL-1 receptor
IL-1RAcP: IL-1 receptor accessory protein
IFNγ: interferon γ
ILC: innate lymphoid cell
iNKT: invariant Natural Killer T
IR: ischemia-reperfusion
IRS-1: insulin receptor substrate-1
KC: Kupffer cells
LSEC: liver sinusoidal endothelial cells
MAPK: mitogen-activated protein kinases
miR: microRNA
NAFLD: non-alcoholic fatty liver disease
NF-κB: nuclear factor- κB
NLRP3: NOD-like receptor family, pyrin domain containing 3
NLRP6: NOD-like receptor family, pyrin domain containing 6
NAPQI: N-acetyl-*p*-benzoquinone-imine
NK: natural killer
PAMP: pathogen-associated molecular pattern
PMN: polymorphonuclear neutrophils
PR3: proteinase 3
ROS: Reactive oxygen species
SIGIRR: single Ig IL-1 related receptor
ST2L: suppression of tumorigenicity 2 ligand
sST2: soluble ST2
TAM: Tumor-associated macrophages
TCR: T-cell receptor
Th: T helper
TLR: toll-like receptor
TLR/IL-1R: TIR domain TLR/IL-1R
TNF: tumor necrosis factor
Treg: T regulatory
TXNIP: thioredoxin-interacting protein
WT: wild-type.
